# Supplementary material for: Chemometrics-aided surface-enhanced Raman spectrometric detection and quantification of GH and TE hormones in blood
Source: PLoS One. 2025 May 23;20(5):e0323697. doi: 10.1371/journal.pone.0323697 (PMC12101856; doi:10.1371/journal.pone.0323697)
Supplement: Attachment — Submitted filename: Reviewercomments_letter2.pdf [file pone.0323697.s001.pdf]

Ondieki Moraa Annah,
Department of Physics,
Faculty of Science and Technology,
University of Nairobi,
P.O. Box 30197-00100,
Nairobi-Kenya.
January 8th, 2025.

To the PLOS ONE Journal Editorial team,
Dear Sir/Madam,

RE: Response to Reviewers for Manuscript Number: PONE-D-24-37774R1
We appreciate the opportunity to submit a revised version of our manuscript titled "Chemometrics-aided Surface-enhanced Raman spectrometric detection and quantification of GH and TE hormones in blood" to the PLOS ONE Journal. The time and effort you and the reviewers put into offering your insightful comments on our manuscript are greatly appreciated. We appreciate the reviewer's illuminating remarks on our paper. Most of the reviewer's suggestions have been incorporated into the adjustments we've made. The manuscript's changes have been highlighted. The reviewers' comments and concerns are addressed in detail here.
Minor issues:

Comment 1: "These model architectures were determined through experimentation, using empirical testing and optimization to balance accuracy and efficiency." --------- Was there cross validation? If not, will this cause model selection bias?

Response: Yes, we employed cross-validation during the model selection process to mitigate the risk of model selection bias. Specifically, we used k-fold cross-validation to evaluate the model's performance on multiple subsets of the data.

Comment 2: Response to the original comment #3 "By taking several spectra i.e., 30 spectra on each sample then perform PCA." ----------- Which data would be used to perform PCA? If 30 spectra from the new sample is used to perform PCA, the eigenvectors (loadings) obtained from the 30 spectra of the new sample may be very different from those used to train the model. Describe how the data was pre-processed.

Response: Thanks for pointing out this. Before applying PCA, the spectral data underwent Baseline Correction to remove low-frequency background variations; Smoothing using Savitzky-Golay filter to reduce high-frequency noise while preserving the spectral features; Normalization to maximum intensity to account for variations in absolute signal intensity (described in details in Ondieki et al., 2023).

In this study, PCA) is used as part of the preprocessing and feature extraction pipeline to reduce the dimensionality of spectral data. PCA was applied to the training dataset, which consists of spectra from a wide range of samples, to derive the eigenvectors (principal components or loadings) that best capture the variance across the dataset. For sure using 30 spectra from the new

sample directly to compute PCA eigenvectors is not recommended because it could lead to eigenvectors that are inconsistent with the established model. Instead, the new sample’s spectra are processed as described above and then projected onto the PCA model trained on the broader dataset. This approach ensures that the interpretation of the new sample is consistent with the trends observed in the training data.

We look forward to hearing from you in due time regarding our submission and to answering any additional inquiries and comments you might have.

Kind Regards,

Annah Moraa,
moraa94annah@gmail.com
