# Supplementary material for: Chemometrics-aided surface-enhanced Raman spectrometric detection and quantification of GH and TE hormones in blood
Source: PLoS One. 2025 May 23;20(5):e0323697. doi: 10.1371/journal.pone.0323697 (PMC12101856; doi:10.1371/journal.pone.0323697)
Supplement: Attachment — Submitted filename: Reviewercomments_letter1.pdf [file pone.0323697.s002.pdf]

Ondieki Moraa Annah,
Department of Physics,
Faculty of Science and Technology,
University of Nairobi,
P.O. Box 30197-00100,
Nairobi-Kenya.
April 6th, 2025.

To the PLOS ONE Journal Editorial team,
Dear Sir/Madam,

RE: Response to Reviewers for Manuscript Number: PONE-D-25-09375

We appreciate the opportunity to submit a revised version of our manuscript titled "Chemometrics-aided Surface-enhanced Raman spectrometric detection and quantification of GH and TE hormones in blood" to the PLOS ONE Journal. The time and effort you and the reviewers put into offering your insightful comments on our manuscript are greatly appreciated. We appreciate the reviewer's illuminating remarks on our paper. Most of the reviewer's suggestions have been incorporated into the adjustments we've made. The manuscript's changes have been highlighted. The reviewers' comments and concerns are addressed in detail here.

Major issues:

Comment 1: Thank you for stating the following financial disclosure: “Swedish International Development Cooperation Agency (SIDA) through the International Science Programme (ISP), Uppsala University, KEN:04, Prof. Kenneth A. Kaduki” Please state what role the funders took in the study. If the funders had no role, please state: "The funders had no role in study design, data collection and analysis, decision to publish, or preparation of the manuscript." If this statement is not correct you must amend it as needed.

Response: The funders had no role in study design, data collection and analysis, decision to publish, or preparation of the manuscript

Comment 2: Thank you for stating the following in the Acknowledgments Section of your manuscript: “We sincerely express our gratitude to the Swedish International Development Cooperation Agency (SIDA) through the International Science Programme (ISP), Uppsala University, for sponsoring this research.” We note that you have provided funding information that is currently declared in your Funding Statement. However, funding information should not appear in the Acknowledgments section or other areas of your manuscript. We will only publish funding information present in the Funding Statement section of the online submission form.

Response: Removed from acknowledgement section as advised.

Reviewer#1

This manuscript presents a quantitative detection of hormones using SERS with a portable Raman spectrometer. The SERS spectra of whole blood from rats injected with hormones are distinguished from those of control samples (without hormone injection) through chemometric analysis. Furthermore, the authors examine temporal variations at different time points following hormone injection. Notably, hormone concentrations are predicted via SERS using an artificial neural network (ANN) model, and the temporal trends appear to be roughly consistent with those obtained through ELISA. The rapid detection of hormones is of significant importance, as it pertains to health monitoring, doping control, and related applications. However, there may be statistical issues. Therefore, I can recommend publication after resolving the following concerns.

Major issues:

Comment 1: Fig. 1: Are these spectra averaged? If so, the number of spectra used to obtain the average should be specified. Additionally, are these spectra normalized? If so, this should be explicitly stated.

Response: Thanks for pointing out this. We have included as suggested.

Comment 2: Lines 189–192: Does the observed difference exceed the spectral deviation? A statistical analysis should be conducted to confirm this.

Response: We appreciate for the insightful suggestion. Descriptive statistics and one-way ANOVA, has been done to confirm this.

Comment 3: Fig. 4: The 3D plots provide limited clarity in distinguishing the temporal variations among (a)–(d). To enhance interpretability, corresponding 2D plots should also be provided.

Response: We appreciate your suggestion to include 2D plots for better clarity in distinguishing temporal variations. However, upon further analysis, we found that 2D plots do not provide significant additional segregation of the data, as the overlapping regions obscure key trends that are better visualized in the 3D representations.

Minor issues:

Comment 1: Section 2.2: The fabrication method or source of AgNPs should be specified.

Response: Included.

Comment 2: Lines 88–89: "30" and "2" -> "Thirty" and "Two."

Response: Corrected.

Comment 3: Lines 123, 125: The meaning of the asterisks should be clarified. Are they needed?

Response: We have corrected this.

Comment 4: Lines 162–164: These sentences can be integrated into the following paragraph due to thematic continuity.

Response: Combined as suggested.

Comment 5: Lines 199–201: These sentences can be incorporated into the next paragraph, as they address a related topic.

Response: Done as suggested.

Comment 6: Lines 242–244: This paragraph can be merged with the preceding one due to their shared subject matter.

Response: Combined.

Comment 7: Lines 245–246: This paragraph can be combined with the following one for coherence.

Response: Combined as suggested.

Comment 8: Line 271: A new paragraph can begin here, and the sentences up to line 275 can be merged with the subsequent paragraph, as they introduce and expand upon a related topic.

Response: Done as suggested.

Comment 9: Lines 296–302: This paragraph can be combined with the following one for improved cohesion.

Response: Done.

Reviewer#2

Comment 1: The abstract needs to be re-phrased. For example, sentences like, "Current methods of detecting them suffer from being costly, time-consuming, non-portable and others." Then, "Secondly, use of artificial neural network (ANN) models in predicting levels of these hormones in blood". Do these sentences look complete? Again, "This work explored, first, Surface-Enhanced Raman Spectroscopy (SERS)....." does not sound professional. I would appreciate it if the authors could re-phrase and reorganize these few lines. Also, I expect the authors must adopt a more professional approach in writing the abstract.

Response: We greatly appreciate your constructive feedback and suggestions for improving the abstract. Based on your comments, we have rephrased and reorganized the abstract.

Comment 2: In the Introduction section, "The primary methods currently used to measure these hormone levels in blood are currently based on immunoassays and mass spectrometry as described in (5)". Can the authors kindly elaborate here? Include a few more references and talk about why SERS is advantageous as compared to other techniques/approaches.

Response: Thanks for pointing out this. We have done as suggested.

Comment 3: In Fig. 1, rats injected both GH only....rats injected both TE only... Remove both.

Response: We appreciate your suggestion to remove the GH and TE injections from Fig. 1. However, our intention was to explore the potential combined effects of both GH and TE injections, alongside the individual effects of each hormone. By including both injections, we aimed to observe if any distinct biochemical changes emerge when both hormones are administered together, which would not be evident from the individual hormone injections alone. Therefore, we have retained the GH and TE injections together in Fig. 1 to reflect this comparative aspect of our study.

Comment 4: For Figs. 1, 3 and 5, the Raman spectrum for different conditions cannot be easily distinguished. Although for Fig.1 it's not that bad, however, for Figs.3 and 5, the individual spectrum/peaks are not clearly visible. It is advisable to vertically shift the individual spectrum for better clarity.

Response: We appreciate your feedback and suggestion to vertically shift the individual spectra in Figures. While we understand the concern, we believe that the current presentation of the spectra effectively conveys the necessary information, as the variations in intensity and spectral features are still distinguishable, especially in Fig. 1. For Figs. 3 and 5, the overlap of individual spectra is a result of the subtle biochemical changes we are investigating, and the vertical shift may not significantly improve the visibility of the spectral differences. We instead enhanced the figure clarity by using different line colors.

Comment 5: "The SERS spectra of blood from non-injected rats and the injected, as shown in Fig 1, are nearly identical, with a few minor band intensity variations suggesting subtle but distinct biochemical changes....". It is good to explain/justify why there is not much change/shift observed between the non-injected and injected spectra. What do the authors mean by subtle but distinct biochemical changes?

Response: We appreciate your insightful comment. To clarify this point, we have revised the text in Section 3.1.

Comment 6: The authors must try to enhance the overall readability of the paper to ensure it is accessible to a broad audience. Also, please check the reference formatting.

Response: Done as suggested.

We look forward to hearing from you in due time regarding our submission and to answering any additional inquiries and comments you might have.

Kind Regards,

Annah Moraa,
moraa94annah@gmail.com
